# A deep learning framework to scale linear facial measurements to actual size using horizontal visible iris diameter: a study on an Iranian population

**DOI:** 10.1038/s41598-023-40839-6

**Published:** 2023-08-23

**Authors:** Zeynab Pirayesh, Sahel Hassanzadeh-Samani, Arash Farzan, Mohammad Hossein Rohban, Mohammad Soroush Ghorbanimehr, Hossein Mohammad-Rahimi, Saeed Reza Motamedian

**Affiliations:** 1 Department of Orthodontics and Dentofacial Orthopedics, School of Dentistry, Zanjan University of Medical Sciences, Zanjan, Iran; 2 Topic Group Dental Diagnostics and Digital Dentistry, ITU/WHO Focus Group AI on Health, Berlin, Germany; 3 Dentofacial Deformities Research Center, Research Institute of Dental Sciences, Shahid Beheshti University of Medical Sciences, Tehran, Iran; 4 Computer Engineering Department, Sharif University of Technology, Tehran, Iran; 5 Department of Computer Sciences and Software Engineering, Concordia University, Montreal, Canada; 6 Postdoc Research Fellow, Division of Artificial Intelligence Imaging Research, University of Maryland School of Dentistry, Baltimore, Maryland USA

**Keywords:** Dentistry, Computer science, Health care

## Abstract

Digital images allow for the objective evaluation of facial appearance and abnormalities as well as treatment outcomes and stability. With the advancement of technology, manual clinical measurements can be replaced with fully automatic photographic assessments. However, obtaining millimetric measurements on photographs does not provide clinicians with their actual value due to different image magnification ratios. A deep learning tool was developed to estimate linear measurements on images with unknown magnification using the iris diameter. A framework was designed to segment the eyes’ iris and calculate the horizontal visible iris diameter (HVID) in pixels. A constant value of 12.2 mm was assigned as the HVID value in all the photographs. A vertical and a horizontal distance were measured in pixels on photographs of 94 subjects and were estimated in millimeters by calculating the magnification ratio using HVID. Manual measurement of the distances was conducted on the subjects and the actual and estimated amounts were compared using Bland–Altman analysis. The obtained error was calculated as mean absolute percentage error (MAPE) of 2.9% and 4.3% in horizontal and vertical measurements. Our study shows that due to the consistent size and narrow range of HVID values, the iris diameter can be used as a reliable scale to calibrate the magnification of the images to obtain precise measurements in further research.

## Introduction

Facial soft tissue analysis plays an essential role in diagnosis and treatment planning in various medical disciplines, such as plastic surgery^1^, maxillofacial surgery^2^, orthodontics^3^, and prosthodontics^4^. Facial features are not only associated directly with aesthetics but can also be indicators of congenital and developmental abnormalities in the oromaxillofacial region^5^. Considering the advantages of indirect assessments of facial soft tissue (e.g., photogrammetry) compared to direct manual measurements on the patients, most clinicians prefer the former^6^. Photogrammetric methods need less chairside time, are less aggressive and more acceptable to the patient, and provide a permanent record that can be used as a reference in later stages of the treatment^7^. Conducting various linear measurements on the patient’s face allows clinicians to diagnose discrepancies such as asymmetry or growth abnormalities, thus enabling them to plan the best possible course of treatment accordingly^8^.

An obstacle we encounter while performing linear measurements on photographs is scaling them to actual sizes^9^. Although clinical facial photography should follow some standard rules (e.g., adequate lighting and adjusting the patient’s posture to neutral head position (NHP)), the photograph magnification will differ depending on various variables. When an image is captured of an object, depending on the camera's features (e.g., the focal length of the lens) and the photographic technique (e.g., the distance between the camera and the object), the resulting photograph can be smaller or larger than the object itself^10^. Therefore, measuring the distance between two facial landmarks on the facial photograph would not provide us with the actual measurement of that distance on the face. As a result, the indirect assessment of the linear measurements would be relatively inaccurate. One of the ways to overcome this problem is to place a ruler next to the face while capturing the photograph for later calibration. However, it is not commonly used in routine clinical facial photography^11^.

Nowadays, with advancements in technology, the world is moving towards automation: developing automatic tools to perform particular activities without the need for human resources^12^. One of the tools used frequently in medicine over the past decade is artificial intelligence (AI): systems that can mimic humans’ deduction process and solve a problem as a person would^13^. Deep learning is one of the branches of AI popularly employed in image analyses^14^. The multi-layer structure of deep learning algorithms results in a better performance in image processing. Thus, these frameworks have been utilized in various medical subfields assisting clinicians in diagnosis, treatment planning, and prognosis prediction^15^.

Although employing AI for scaling facial photographs to the actual size has not been done before, studies have been conducted to overcome the size estimation problem in other fields using deep learning^16–19^. In a study, the authors estimated the length of a specific type of fish by developing a statistical model using the ratio of the head's length to the overall length of that particular kind of fish^16^. In another study, a deep learning model was utilized to classify polyps in colonoscopy images into two categories of more and less than 10 mm in diameter by estimating the depth of the image^17^. Another method used in studies was to use deep learning techniques to estimate the size of an object by using a specific item as a “scale” in the image^18^. For instance, Apolo-Apolo et al., used a wooden stick as a reference object in the photographs of citrus trees. By knowing the exact size of the wooden stick, the estimation of different measurements (such as fruit size) will be possible using a “calibration process”^19^. In this process, by calculating the ratio of the reference object’s length in centimeters to its length in pixels, we will be able to estimate the actual size of any object in the picture by multiplying the object’s length in pixels by the calculated ratio.

This study aims to develop a deep learning tool to estimate linear measurements on facial photographs with unknown magnification. For this purpose, we scaled horizontal and vertical linear measurements to their actual sizes using horizontal visible iris diameter (HVID). Studies state that HVID falls within a particular and relatively small range of approximately 10–13 mm^20,21^. When assessed within a specific race, the standard deviation is expected to be less than 10% of the average^22,23^. Considering the small range of standard deviation values, a constant value can be assigned to HVID in most of the patients. As a result, we would be able to use HVID as a reference object to scale other linear measurements to their actual amounts. Although this method was previously used in facial analysis researches^9,24^, we aimed to automize the process and improve its outcome by implementing deep learning techniques. As a result, a faster and more reliable method would be available to normalize the magnification of frontal-view facial photographs. The deep learning tool presented in this study automatically segments the eye's iris and scales the photograph according to the obtained HVID.

## Materials and methods

### Study design

**Figure 1:**
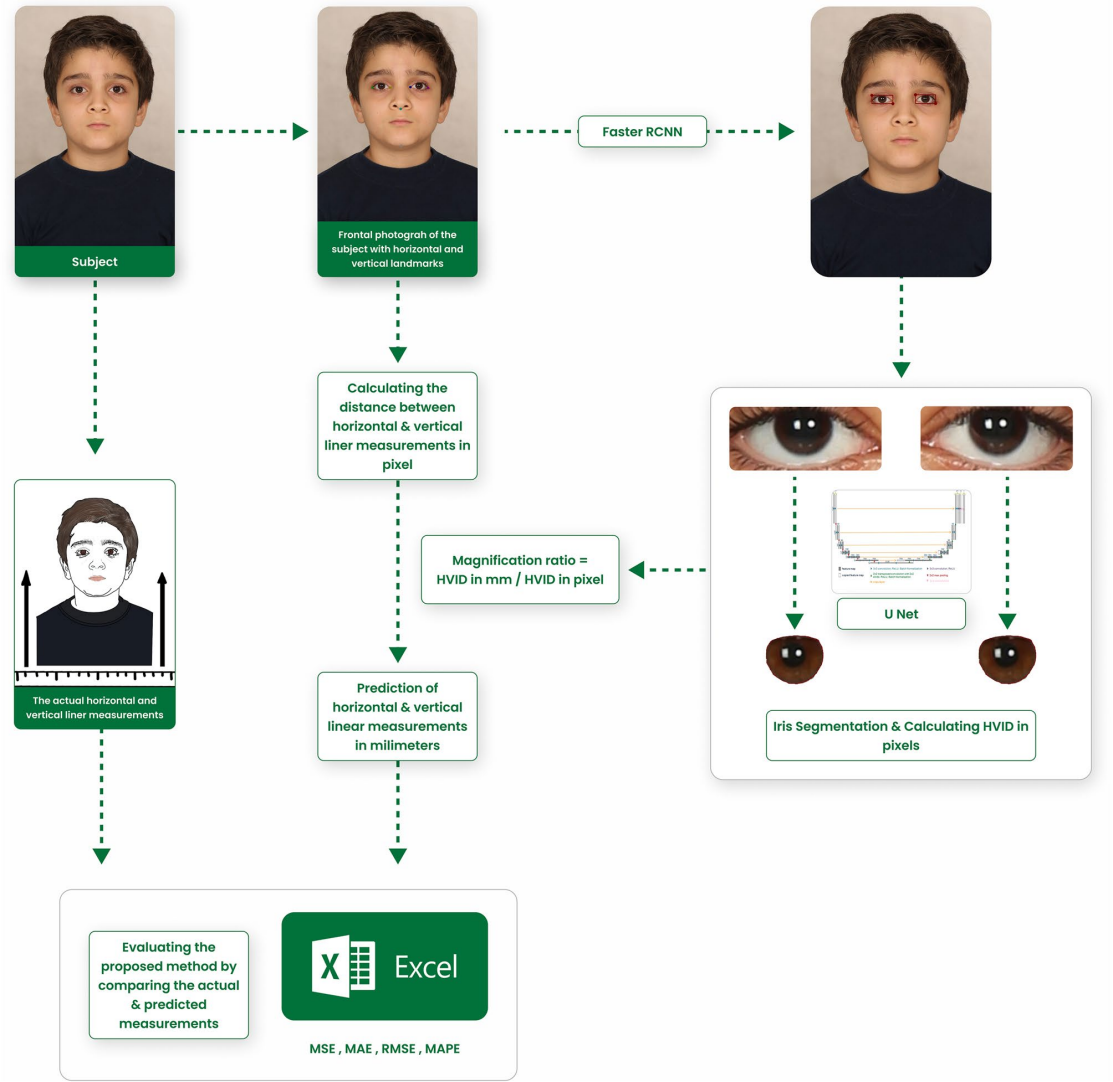
The framework of the proposed method.

The proposed Checklist for Artificial Intelligence in Medical Imaging was adopted for reporting this study^25,26^. Frontal view facial images were collected from the patients of a private clinic. Additionally, two linear measurements were manually obtained from each patient using a caliper. These two distances were also measured in pixels on the obtained photographs. An object detection model and a segmentation model were developed to precisely segment the eyes’ iris. Afterward, the diameter of the iris was measured in pixels, and a magnification ratio was calculated by dividing the iris diameter in millimeters by its diameter in pixels. The value assigned to the iris diameter was 12.2 mm in every patient. The linear measurements in pixels were multiplied by the obtained ratio to predict their actual amount in millimeters. Finally, the predictions were compared to the manual measurements to assess the validity of the technique (Fig. 1). The protocol and procedures used in the study were ethically reviewed and authorized by the ethics committee of the local university (Ethical approval code: IR.ZUMS.REC.1400.395).

### Data sampling

A total of 344 2-dimensional (2D) facial frontal-view photographs (84 male, 260 female/12–45 years old) were obtained from a private orthodontic clinic in Zanjan, Iran. All the photographs were taken with a digital single-lens reflex camera (Canon EOS 5D Mark III, Japan) and according to the inclusion criteria. The photographs used in this study have dimensions of 2362 pixels × 3543 pixels and a horizontal and vertical resolution of 300 dpi. Two hundred and fifty images were used to train the model, and 94 were used to test the automated estimation with the manual measurements. Patients were provided with a written consent form asking permission to use their photographs for training and testing our algorithms.

The inclusion criteria for the photographs were:

- Straight posture and head in neutral head position (NHP)
- One colored, white, or off-white background without any shadow on the face
- Eyes looking straight forward
- Visible ears

The exclusion criteria for the photographs were:

- Blurred photographs
- Patients with any problems related to the eyes, like strabismus
- Patients wearing glasses or colored contact lenses
- Patients below the age of 12

### Preprocessing

Contrast-limited adaptive histogram equalization (CLAHE) is a histogram-based image enhancement method that limits contrast amplification to reduce noise^24^. This algorithm views the histogram as small tiles and operates on each tile separately, which allows for a contrast enhancement with minimum noise amplification^27^. As a result, minimum data would be lost in the process of contrast enhancement, which can especially be significant in medical data^28^. To prepare the images for the networks, they were resized to 256*256. In our work, the inclusion of CLAHE has aided in enhancing the overall accuracy rate.

### Ground truth

To achieve the study's goal, we needed a framework to detect the eyes and generate a mask that surrounds each iris separately. The following steps were taken to prepare the training data: the first step was manually labeling each eye’s region in frontal photographs. This step was conducted by drawing rectangles around the eyes by one of the dentists using LabelMe software (the MIT Computer Science and Artificial Intelligence Laboratory, Cambridge, Massachusetts, USA). The other dentist double-checked the boxes. The upper and lower eyelids were considered as the rectangle's upper and bottom sides, and the canthi of the eyes determined the lateral sides. Since object detection does not require too many samples due to its simplicity, 250 samples were chosen randomly for this step.

**Figure 2:**
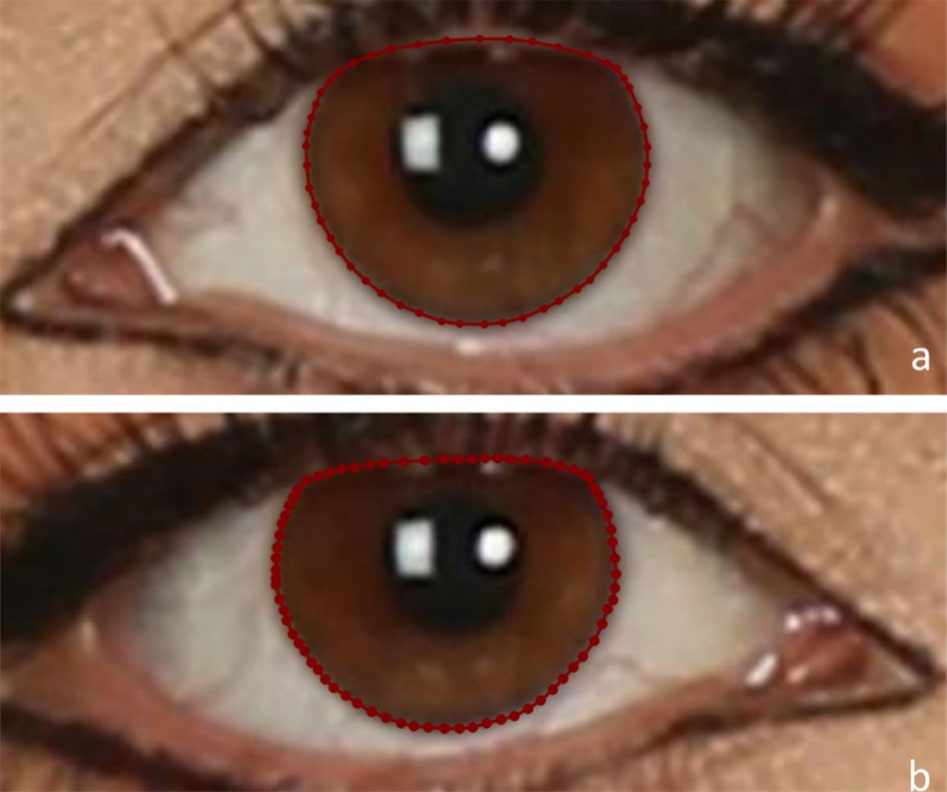
Manually segmented iris, (**a**) left side, (**b**) right.

In the next step, the eyes’ irises were manually labeled by drawing polygons around the contour line of the colored part of all the samples (500 eyes). Two dentists were calibrated and conducted the labeling of the training dataset independently by the LabelMe software. The final segmentation mask was obtained using the intersection of the two annotations. This segmentation step helped calculate the HVID (Fig. 2).

To evaluate the validity of using HVID to estimate the millimeter measurements in the frontal-view facial photographs, the distance between two horizontal landmarks (lateral canthi of the eyes) and two vertical landmarks (subnasale and submental) in 94 patients were measured using a caliper (Dasqua Monoblock Vernier 0–200 mm/0–8′′ Caliper Series 1120-3120 Stainless Steel. Lodi, Italy). Lateral canthi of the eyes are identified as the lateral points where superior and inferior eyelids meet. The subnasale point is defined as the deepest midline point where the base of the nasal columella meets the upper lip, and the submental point is determined as the most inferior midline point of the soft tissue chin^29^. The manual measurements were conducted by a trained dentist with the supervision of an orthodontist. Additionally, the mentioned landmarks were labeled on the photographs of the same 94 patients using LabelMe software, and the distances between two vertical and two horizontal landmarks were computed in pixels (Fig. 1). Then, the distances between the landmarks in the photos were estimated in millimeter. This estimation was performed by calculating a magnification ratio using the obtained amount of HVID in pixels and the average iris diameter in millimeter (Fig. 1). We used different mean values of the iris diameter in this study (from 10.5 to 13.5 mm) and compared the obtained error. The accuracy of scaling based on HVID was assessed by comparing the clinical measurements to those estimated using our calibration method.

### Data partitions

In the first step, 250 frontal-view facial images were fed into an eye detection model (Faster R-CNN). This dataset was divided into 80% training data and 20% test data. For the next step, the 500 extracted ROIs were fed into the segmentation model. These eye images were randomly divided between the training, validation, and test sets using a cross-validation technique (80% training set, 10% validation set, 10% test set). A five-fold cross-validation procedure was performed to assess the model's overall performance. Five separate U-net models were initialized, trained, and validated on a unique training and validation combination. Each fold produces a model that can predict the iris boundary.

### Model

#### Object detection

Several object detection models were assessed for detecting the eyes in the photographs. A Faster Region-based Convolutional Neural Network model (Faster R-CNN) based on ResNet 101 was employed due to its superior performance. Faster R-CNN is a two-stage object detection framework widely used in researches concerning medical imaging. This algorithm combines a region proposal network (RPN) with a convolutional neural network (CNN) to accurately localize objects in an image and has shown desirable outcomes. Rectified linear unit (ReLU) activation function was selected for the network.

#### Segmentation

To achieve the goal of our research, we built a seven-level hierarchy U-net^30,31^. The input starts with three channels of 256 × 256-pixel images. Rectified linear unit (ReLU) activation function in the U-net allows for an equal updating of the weights throughout the model, leading to faster convergence. Additionally, Sigmoid activation function was selected for the final layer.

### Training

For the object detection model, the Adam algorithm was chosen as the optimizer to minimize the loss function. We used a learning rate of 10^–2^, and the default Adam parameters β1 = 0.9, β2 = 0.999, and *decay* = 0. Additionally, sum of cross-entropy loss for region proposals and smooth L1 loss for bounding box regions were selected.

The Adam algorithm was selected as the optimizer for the segmentation model as well. A learning rate of ${10}^{-3}$ and the same default Adam parameters were used. A loss function based on the mean squared error (MSE) between the iris mask and the iris was chosen. The model architecture’s setup and the optimization process were carried out using the deep learning library TensorFlow (2.8.0) and Python (3.10) programming language. The training procedure was performed on a Tesla K80 with 12GB of GDDR5 VRAM, Intel Xeon Processor with two cores @ 2.20 GHz, and 13 GB RAM.

### Evaluation

Our models were evaluated using the test set, and the performance metrics for the best-trained model were reported. These metrics include Precision^32^, Recall^32^, Intersection over Union (IoU)^33^, and Dice coefficient^34^ for the segmentation model and mean Average Precision (mAP)^33^ for the object detection model. The metrics are defined below:

$$Precision\, = \,\frac{TP}{{TP + FP }},\;\;Recall\, = \,\frac{TP}{{TP + FN}},$$

$$Dice\; \, coefficient\, = \,\frac{2*TP}{{\left( {TP + FP} \right) + \left( {TP + FN} \right)}},\;\;IoU\, = \;\frac{TP}{{TP + FP + FN}},$$

$$mAP\; = \;\frac{1}{N} \mathop \sum \limits_{{Recall_{i} }} AP\left( i \right)$$

In the equation, TP, TN, FP, and FN represent the number of true positive, true negative, false positive, and false-negative samples, respectively.

### Statistical analysis

The accuracy of our size estimation method was assessed with the following error analysis indicators: mean absolute error (MAE)^35^, mean squared error (MSE)^35^, root mean square error (RMSE)^35^, and mean absolute percentage error (MAPE)^36^.

The mentioned five indicators are calculated using the equations below, where Ai is the actual value, Pi is the predicted value, and n is the total sample number:

$$MAE = \frac{1}{n}\mathop \sum \limits_{i = 1}^{n} \left| {Ai - Pi} \right|$$

$$MSE = \frac{1}{n}\mathop \sum \limits_{i = 1}^{n} (Ai - Pi)^{2}$$

$$RMSE = \sqrt {\frac{{1\mathop \sum \nolimits_{i = 1}^{n} (Ai - Pi)^{2} }}{n}}$$

$$MAPE = \frac{1}{n}\mathop \sum \limits_{i = 1}^{n} \left| {\frac{{\left( {Ai - Pi} \right)}}{Ai}} \right|$$

MAE shows the magnitude of the error and might be considered a more natural and explicit index compared to RMSE^35^. The more accurate the predictions are, the lower the metrics will be. Bland Altman Plots (BAP) were also used to visually analyze the difference between the manual measurements and AI predictions. All calculations were carried out using Microsoft Excel (Microsoft Corporation (2013)) and Python programming language (Python Software Foundation. Python Language Reference, version 3.10. Available at http://www.python.org).

### Ethics approval and consent to participate

The study was conducted according to the guidelines of the Declaration of Helsinki and approved by the Ethics Committee of Zanjan University of Medical Sciences (Ethical approval code: IR.ZUMS.REC.1400.395, Date of approval: 2021/12/15). Written Informed consent was obtained from all subjects involved in the study.

### Consent for publication

Written informed consent has been obtained from the patient’s guardians (patient in Fig. 1) to publish this paper. A blank form used to obtain consent from the patients is uploaded. This form is written in the writers’ and patients’ native language.

## Results

### Data and statistical analysis

**Figure 3:**
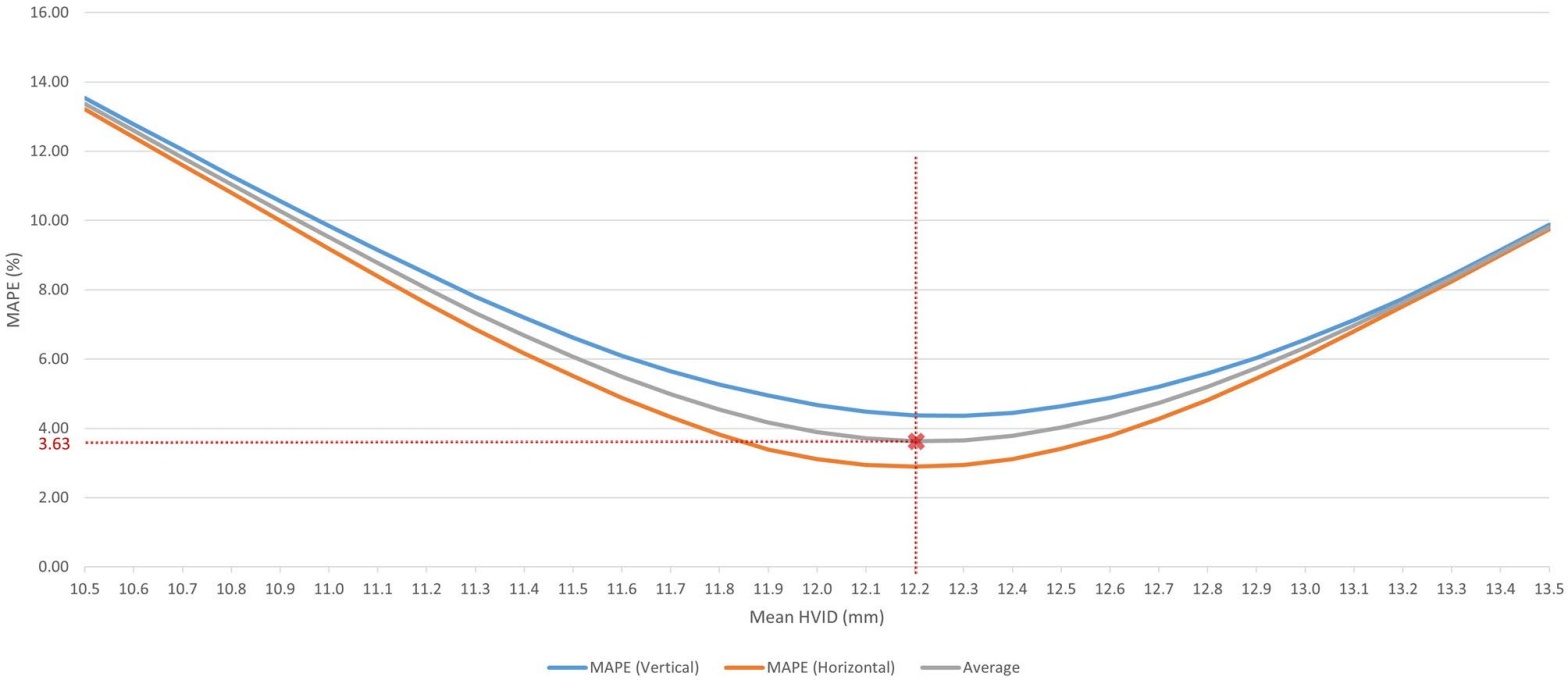
Calculated MAPE for horizontal (lateral canthi distance) and vertical (subnasale-submental distance) measurements according to the assigned HVID.

Of the 344 frontal-view facial photographs that met the eligibility criteria, 250 (65 male, 185 female/13–45 years old) were used to train the object detection and iris segmentation models. The remaining 94 images (19 male, 75 female/12–43 years old) were used to evaluate the difference between actual and predicted linear measurements. These measurements were predicted by using HVID as a scaling reference. Figure 3 demonstrates the obtained MAPEs according to the assigned HVID. The findings indicated that a mean HVID of 12.2 mm would yield the optimum outcomes. This amount was reported in Chen et al. study^37^. Table 1 summarizes the range, standard deviation, and mean of actual and predicted measurements for each dimension.

**Table 1:** The range, mean, standard deviation, and p-value of actual and predicted measurements for horizontal (lateral canthi distance) and vertical (subnasale-submental distance) measurements.

Metric		Range	Mean	SD	P-value
Age		12–43	23.35	7.76	
Subnasale-submental distance	Real	47.25–86	69.11	5.45	0.812
	Predicted	55.53–96.34	68.89	7.15	
Lateral canthi distance	Real	88–110.7	98.06	4.78	0.769
	Predicted	87.26–112.94	97.85	5.02	

**Figure 4:**
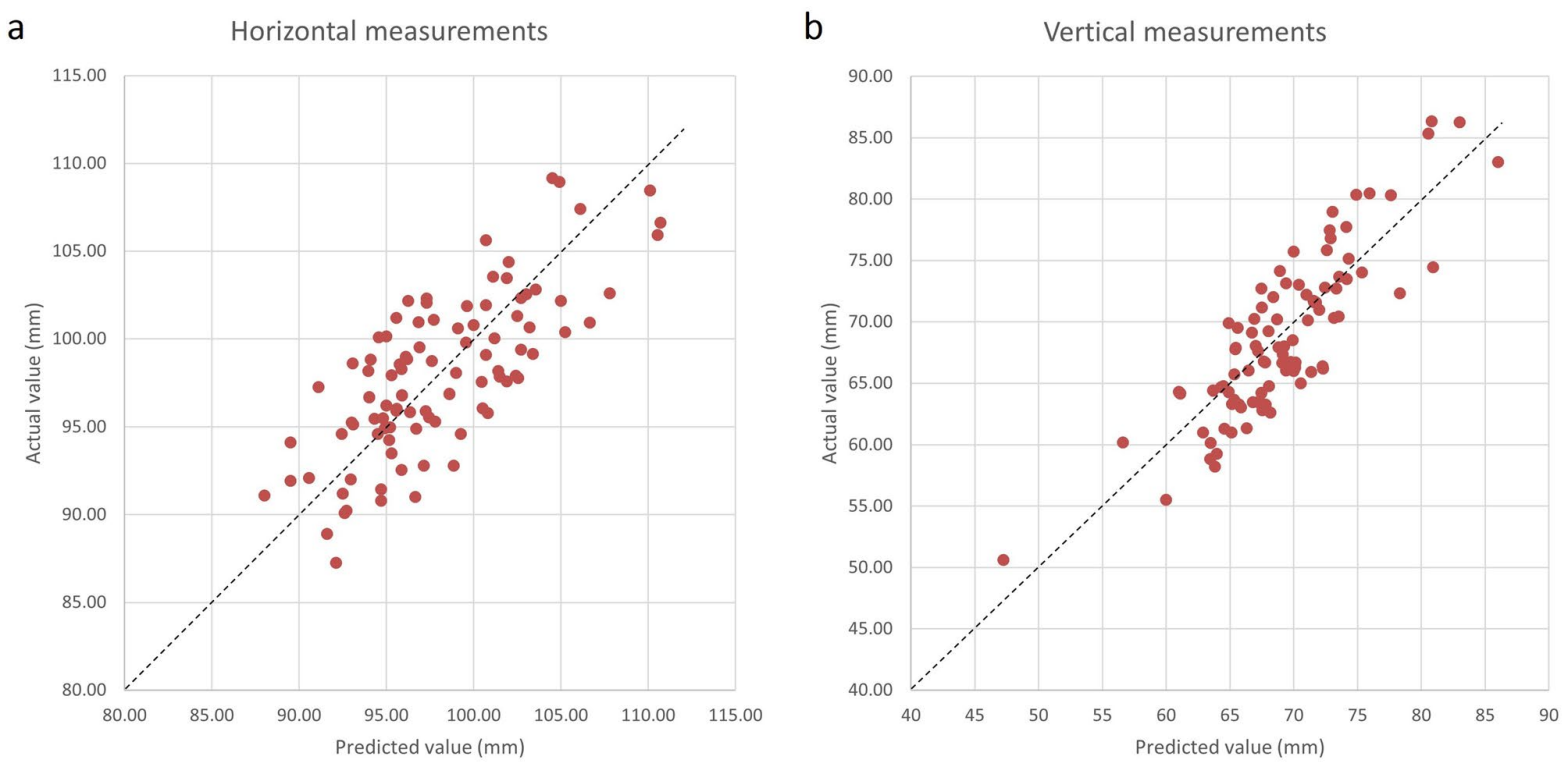
The distribution of predicted and actual (**a**) horizontal (lateral canthi distance) and (**b**) vertical (subnasale-submental distance) measurements. The dashed line represents the zone in which the actual and predicted measurements have the same value.

The results demonstrated that the model’s prediction errors were acceptable, with an average MSE of 11.59 ± 10.84 mm, an average RMSE of 3.40 ± 1.75 mm, an average MAE of 2.93 ± 1.75 mm, and an average MAPE of 3.36 ± 2.15% for the horizontal (2.9%) and vertical (4.3%) measures (Table 2). Also, the actual and predicted values and their difference can be visually evaluated in Fig. 4. The diagrams represent the horizontal and vertical measurements on each data as dots regarding their actual and predicted amounts.

**Table 2:** Prediction errors: MSE, RMSE, MAPE, MAE, and LOA for predicted and actual measurements.

Metric		Horizontal (Lateral canthi distance)	Vertical (subnasale−submental distance)
MSE ± SD	Female	10.38 ± 10.36	11.31 ± 10.58
	Male	13.41 ± 11.61	15.70 ± 12.44
RMSE ± SD	Female	3.22 ± 1.68	3.36 ± 1.76
	Male	3.66 ± 1.85	3.96 ± 1.79
MAPE ± SD	Female	2.84 ± 1.73	4.25 ± 2.58
	Male	3.14 ± 1.84	4.83 ± 2.44
MAE ± SD	Female	2.76 ± 1.68	2.87 ± 1.76
	Male	3.19 ± 1.85	3.56 ± 1.79
Upper LOA (Bias − 1.96*SD)		6.60	7.30
Lower LOA (Bias + 1.96*SD)		− 6.46	− 6.34

Bias is the average of the difference between actual and predicted numbers.

Supplementary Figure S1 illustrates the Bland Altman Plots for horizontal and vertical measurement. The x-axis represents the average of the two actual and predicted values, while the y-axis represents the difference between the two values. The dashed lines show the upper and lower limit of Agreement (LOA), and the solid line is the indicator of the mean difference in the sample. Table 2 shows the exact values of the Bland–Altman analysis.

### Model performance

For the object detection task, the Faster-RCNN model performed best on our dataset. The 250 samples were divided randomly into training set (54 male, 146 female/15–45 years old) and test set (11 male, 39 female/13–33 years old). The batch size was set to 4, and the model was trained for 100 epochs. Additionally, the Adam optimizer was chosen as the model optimizer with a learning rate value of 0.01. The algorithm achieved 100% AP50 and 100% AP75 on the validation set. Table 3 shows the Faster-RCNN performance on our data set compared to other object detection models.

**Table 3:** Performance of the object detection models on the validation set.

Model	AP50	AP75
Faster R-CNN (Selected Model)	100	100
Cascade R-CNN	100	90.899
RetinaNet	98.664	88.533
Yolo v4	87.908	82.033
Fast R-CNN	86.27	76.044

For the segmentation task, several models were run before we selected the U-net model for its better performance. Following the hyperparameter tuning, the batch size was set to 32. Also, the Adam optimizer was selected as the model optimizer with a learning rate value of 0.001.

We trained each fold for 100 epochs. Supplementary Fig. S1-a shows the training and validation loss and Supplementary Fig. S1-b shows the accuracy of training and validation in various epochs from one of the folds. The final average accuracy ± standard deviation between all the folds was 99.46 ± 0.2% (test set). The performance of the model on each fold is provided separately in Supplementary Table S1. Among the five folds, the model from the fifth fold performed the best, with the lowest validation loss of 3.8 × 10^–3^. As a result of the inclusion of CLAHE, our study's overall accuracy rate has been enhanced. The performance evaluation of our selected model (U-net) is compared to other segmentation models in Table 4.

**Table 4:** The segmentation models’ mean (± standard deviation) performance on the test set.

Model	Accuracy	Dice coefficient	IoU	Jaccard Distance	Precision	Recall
U-net (Selected Model)	99.46% (± 0.0023)	98.99% (± 0.0040)	99.14% (± 0.0186)	8.5 × 10^–3^ (± 0.0186)	99.40% (± 0.0031)	98.57% (± 0.0079)
Res-U-net	98.71% (± 0.0074)	98.38% (± 0.0079)	98.51% (± 0.0321)	14.8 × 10^–3^ (± 0.0321)	99.01% (± 0.0083)	97.76% (± 0.0102)
Attention U-net	99.03% (± 0.0030)	98.94% (± 0.0058)	99.13% (± 0.0192)	8.6 × 10^–3^ (± 0.0192)	99.03% (± 0.0042)	98.85% (± 0.0056)
DeepLab V3	93.50% (± 0.0267)	93.18% (± 0.0381)	93.72% (± 0.0384)	62.7 × 10^–3^ (± 0.0384)	94.35% (± 0.0193)	99.04% (± 0.0040)

## Discussion

This study aimed to tackle the existing obstacle in determining the magnification of frontal-view facial photographs. Our results show that assigning a constant value to HVID in all subjects to obtain the magnification ratio of the photograph provides reliable outcomes. The primary hypothesis was that HVID could be used as a scale to measure the distances between anatomical structures of the face in facial photographs. This hypothesis is attainable since the accepted normal range for the corneal diameter is usually between 10 and 13 mm^21^. As a result, using the proposed technique on digital images, the distances between landmarks in pixels can be accurately converted to millimeters. Therefore, provided that the eyes are pointed straight forward and the head is in NHP, images with different techniques, sources, and magnifications can be standardized in order to determine the actual amounts of linear facial measurements.

Millimetric measurements are an essential part of facial analysis. One important millimetric measurement is calculating the distance between landmarks and the midline to assess symmetry. Additionally, millimetric measurements may be used to assess vermilion show or lip incompetency. These measurements usually fall in the range of a few millimeters. Thus, the difference between the actual and estimated values would be insignificant, given that the mean error rate of our approach is less than 5%. However, the inaccuracy of 5–10% might be noticeable in greater measurements, even though they are not commonly conducted in facial soft tissue analysis.

The “iris-dependent calibration” technique was primarily proposed in a study conducted by Spörri et al.^24^ in 2004. Their study aimed to evaluate nasolabial angle and nasal tip projection on pre-existing profile photographs of 42 patients. To be able to report the absolute values of the measurements, they defined the iris radius as 5.75 mm in every photograph (iris diameter = 11.5 mm) and calibrated the linear measurements accordingly. Although the accuracy and reliability of the method were not reported in the aforementioned study, the authors claim that their technique is preferable to other methods, such as manual measurements, due to the reproducibility of the results.

In another study in 2010, Driessen et al.^9^ used iris to calibrate images of 100 children between the ages of 5–18 for facial anthropometry in digital photographs. According to their findings, the iris width of 11.22 may be considered a reliable standard to calibrate the magnification of facial structures on the same plane as the forehead. However, a larger iris width should be employed to convert the magnification for parts of the face that are in a deeper plane than the forehead. Additionally, it was demonstrated in their study that there is no gender or age difference in the appropriate size of the iris for converting the magnification of images. The study did not report the error range of the proposed size estimation method.

We assessed the iris-dependent calibration technique in the present study and reported the resulting error range. Our study’s advantage over the previous works is the utilization of deep learning techniques to calculate the HVID. By automizing the calibration process, human error will be minimized, and a more reproducible and reliable result will be achieved.

Previous anthropometric and anatomical studies in different populations reported different sizes for the mean and range of HVID. Chen et al.^37^ investigated HVID using manual calipers (12.22 mm) and automated measurement techniques (12.12 mm) and suggested expanding the upper limit of normal horizontal corneal diameter to 13.2 mm. Claude et al.^38^ reported a corneal diameter of 12.0 ± 0.5 mm from the photographs of young adults. Similarly, Pinero et al.^39^ reported a mean HVID of 12.25 ± 0.49 mm (range 11.34–13.16 mm) in their population. Hashemi et al.^40^ suggested the normal HVID range of 10.76–12.60 mm. The mean HVID we used in this study was 12.2, according to the previously reported HVID ranges. The only longitudinal study, which measured the iris diameters in 13 subjects over a mean age difference of 8.3 years, found a regression equation for the growth of the iris. The mentioned paper suggested that the corneal diameter tends to increase by 0.3 mm sometime between 3 and 4 months and 3–18 years of age, but it does not solve the question of when the growth occurred. However, the writers claim that this growth most likely happens in the first few months of infancy^41^. According to Augusteyn et al.^42^, the cornea's horizontal diameter reaches its maximum within the first 2 years after birth. Müller et al.^43^, Chen et al.^37^, and Cakmak et al.^44^ found no correlation between age and iris diameter, supporting the concept that the iris is fully grown in the first years of life and can be helpful as a fixed reference.

Facial landmarks and their relations can help assess facial asymmetry, discrepancies, and anomalies like hypertelorism and chin or nose deviation^45^. Due to their low cost and ease of storage, digital photographs are ideal for conducting these evaluations^46^. Moreover, a more definite point localization is possible by enlarging the photograph as necessary. However, 2D digital photographs may not be taken under the same conditions and must be standardized regarding magnification. The iris was considered a scale that yielded acceptable findings due to its persistent average size. The slight difference between actual and predicted sizes was highlighted by a MAPE of less than 5%. It seems that AI can replace manual measurements while dissolving disagreements between specialists. The standardization approach offered herein represents precise distance measurements based on iris-dependent calibration, allowing for more accurate facial analysis in future research. The standardization technique may also be applied to other fields like cosmetic surgery. Since few datasets with a scale on images are available, we can benefit from the results of this study to generate models that perform automatic facial frontal analysis using databases containing photos taken without any scale.

The current study had some limitations. First, all the samples were collected from one center and all the participants were from the same ethnicity (Iranian). According to related studies, HVID ranges and mean values can vary between races due to the differences in their overall physique. For instance, a range of 10.76–12.6 mm was reported for the HVID in Iranian population^40^, 10–12 mm in African (Nigerian) population^23^, 11.5–12.3 in Caucasians^47^, and 10.5–12.4 in the Chinese population^48^. Although the differences in the mean values seem small, further studies are encouraged to perform external tests and evaluate the performance of our method using diverse datasets from various populations. This will provide valuable insights into the generalizability and applicability of our findings across different ethnicities and populations. Second, The iris diameter was not adjusted for measuring the distances between landmarks located in different depths of the facial photograph. However, such measurements are less common during facial frontal image analysis. Lastly, the evaluations of the model revealed that adding more samples would not reduce scaling bias. It seems that the concept of considering the iris as a scale results in some unpreventable error. Nevertheless, increasing the sample size in future research may benefit in improving the generalizability of the results and enhancing the reliability of the findings across a broader population.

The proposed method of image scaling can be utilized to expand on automated models of facial analysis, which are used to diagnose and measure asymmetries and other growth discrepancies with acceptable accuracy. Nevertheless, there still is a pending need for further evaluation of this accumulated model's accuracy with the performance accuracy of other similar AI frameworks (image processing tasks), such as Image Depth Estimation tasks and other similar image scaling algorithms. Moreover, since the use of three-dimensional volumetric photography for aesthetic purposes has been on the rise in recent years, this study can be an instigator to further research on the accuracy of the HVID-based estimated linear measurements in a three-dimensional plane.

## Conclusion

The present study proposes a fully automated method based on deep learning techniques to estimate the actual value of linear facial measurements in facial frontal-view photographs with unknown magnification. The presented model automatically segments the iris, measures the horizontal diameter in pixels, and uses it as a reference to scale other horizontal and vertical linear measurements on the photograph to their actual size. Our model showed satisfactory results with Mean Absolute Percentage Error (MAPE) of 2.9% and 4.37% in horizontal and vertical linear measurements. Since the most practical clinical linear measurements on the face are in the range of a few millimeters, the obtained error can be considered insignificant while applying the proposed size estimation method in facial analysis. Therefore, this technique can prove beneficial in clinical investigations of facial esthetic-related treatments by providing clinicians with the actual millimetric value of different linear measurements with minimum error.

### Supplementary Information

Supplementary Information 1.

Supplementary Figures.

Supplementary Table S1.

## Data Availability

The data presented in this study are available on request from the corresponding author. The data are not publicly available due to ethical reasons.
