# Supplementary material for: A deep learning framework to scale linear facial measurements to actual size using horizontal visible iris diameter: a study on an Iranian population
Source: Sci Rep. 2023 Aug 23;13:13755. doi: 10.1038/s41598-023-40839-6 (PMC10447546; doi:10.1038/s41598-023-40839-6)
Supplement: Supplementary file 1 — Supplementary Information 1. [file 41598_2023_40839_MOESM1_ESM.docx]

**به نام خدایی که شفا از اوست**

رضایت نامه شرکت در طرح **«توسعه یک ابزار هوش مصنوعی برای تخمین اندازه‌های خطی در صورت»**

آقا/ خانم محترم

بدین وسیله از شما جهت شرکت در پژوهش فوق الذکر دعوت به عمل می آید. اطلاعات مربوط به این پژوهش در این برگه خدمتتان ارائه شده است و شما برای شرکت یا عدم شرکت در این پژوهش آزاد هستید.

شما مجبور به تصمیم گیری فوری نیستید و برای تصمیم گیری در این باره می توانید سؤالات خود را از تیم پژوهشی بپرسید و با هر فرد صاحب نظری که مایل باشید مشورت نمایید. قبل از امضای این رضایت نامه مطمئن شوید که به نحو کافی و مناسب متوجه تمامی اطلاعات این فرم شده اید و به تمام سؤالات شما پاسخ داده شده است.

در این طرح "عکس صورت از نمای روبه‌رو" در حال بررسی است.

مجری طرح دکتر سعیدرضا معتمدیان/ دانشجو ساحل حسن‌زاده سامانی

1. من آگاه شدم که اهداف این پژوهش عبارتند از:

- توسعه سیستم خودکار برای آنالیز فوتوگرافی صورت از نمای روبه رو
- آنالیز عکس‌های فوتوگرافی صورت از نمای روبه‌رو توسط دو فرد آموزش دیده
- آنالیز عکس آنالیز عکس‌های فوتوگرافی صورت از نمای روبه‌رو توسط سیستم خودکار
- مقایسه میان آنالیزهای انجام شده توسط افراد آموزش دیده و سیستم خودکار

1. من آگاه شدم که شرکت من در این پژوهش کاملاً داوطلبانه است و مجبور به شرکت در این پژوهش نیستم.

به من اطمینان داده شد که اگر حاضر به شرکت در پژوهش نباشم، از مراقبت های معمول تشخیصی و درمانی محروم نخواهم شد. رابطه درمانی من با مرکز درمانی و کادر پزشکی دچار اشکال نشده و روشهای معمول درمان برای من ادامه پیدا خواهد کرد.

1. من آگاه شدم که حتی پس از موافقت با شرکت در پژوهش می توانم هر وقت که بخواهم، از پژوهش خارج شوم. خروج من از پژوهش باعث محرومیت از دریافت خدمات مراقبتی، تشخیصی و درمانی معمول برای من نخواهد شد و مستلزم پرداخت جریمه یا خسارتی نخواهد بود.
2. نحوه ی همکاری اینجانب در این پژوهش به این صورت است:

تهیه عکس از صورت از نمای رو‌به رو

1. منافع احتمالی شرکت اینجانب در این مطالعه به این شرح است:

وجود ندارد.

1. آسیب ها و عوارض احتمالی شرکت در این مطالعه به این شرح است:

وجود ندارد.

1. من آگاه شدم که دست اندرکاران این پژوهش، کلیه اطلاعات مربوط به من را نزد خود به صورت محرمانه نگه داشته و فقط اجازه دارند نتایج کلی و گروهی این پژوهش را بدون نام و مشخصات اینجانب منتشر کنند.
2. من آگاه شدم که هیچ یک از هزینه های انجام مداخلات پژوهشی به شرح ذیل برعهده من نخواهد بود:

عکس‌برداری

1. من آگاه شدم که چنانچه در فرآیند پژوهش تغییری حاصل گردد که بر سلامت و تصمیم گیری من جهت تداوم شرکت در طرح تأثیر گذار باشد به اطلاع من رسانده خواهد شد.
2. من آگاه شدم که کمیته اخلاق در پژوهش های زیست پزشکی به آدرس: پژوهشکده علوم دندانپزشکی، دانشکده دندانپزشکی، دانشگاه علوم پزشکی شهید بهشتی و تلفن 22413897 با هدف نظارت بر رعایت حقوق اینجانب می تواند به اطلاعات من دسترسی داشته باشد.
3. من آگاه شدم اگر اشکال یا اعتراضی نسبت به دست اندرکاران با روند پژوهش داشته باشم می توانم با کمیته اخلاق در پژوهشهای زیست پزشکی به آدرس پژوهشکده علوم دندانپزشکی، دانشکده دندانپزشکی، دانشگاه علوم پزشکی شهید بهشتی و تلفن 22413897 تماس گرفته و مشکل خود را مطرح نمایم.

لذا بدینوسیله اینجانب ..................................... فرزند .................. با شماره شناسنامه ............... و کدملی ..................................... با توجه به موارد بالا و کسب آگاهی کافی، مراتب رضایت آگاهنه و داوطلبانه خویش را جهت شرکت در طرح پژوهشی فوق اعلام می دارم.

نشانی و شماره تماس: .............................................................................................................................................................................................................................................

امضای شرکت کننده و اثر انگشت:

تاریخ:

**شاهد:**

نام و نام خانوادگی:

شماره شناسنامه:

کدملی:

شماره تماس:

امضای شاهد و اثر انگشت:

تاریخ:

اینجانب **دکتر سعیدرضا معتمدیان/ دانشجو ساحل حسن‌زاده سامانی** خود را ملزم به اجرای تعهدات مربوط به مجری در مفاد فوق دانسته و متعهد می گردم در تأمین حقوق و ایمنی شرکت کننده در این پژوهش تلاش نمایم.

مهر و امضای مجری پژوهش

این فرم اطلاعات و رضایت آگاهانه در دو نسخه تنظیم شده و پس از امضاء، یک نسخه در اختیار آزمودنی و نسخه دیگر در اختیار مجری طرح قرار خواهد گرفت.
