# Supplementary material for: A deep learning framework to scale linear facial measurements to actual size using horizontal visible iris diameter: a study on an Iranian population
Source: Sci Rep. 2023 Aug 23;13:13755. doi: 10.1038/s41598-023-40839-6 (PMC10447546; doi:10.1038/s41598-023-40839-6)
Supplement: Supplementary file 2 — Supplementary Figures. [file 41598_2023_40839_MOESM2_ESM.docx]

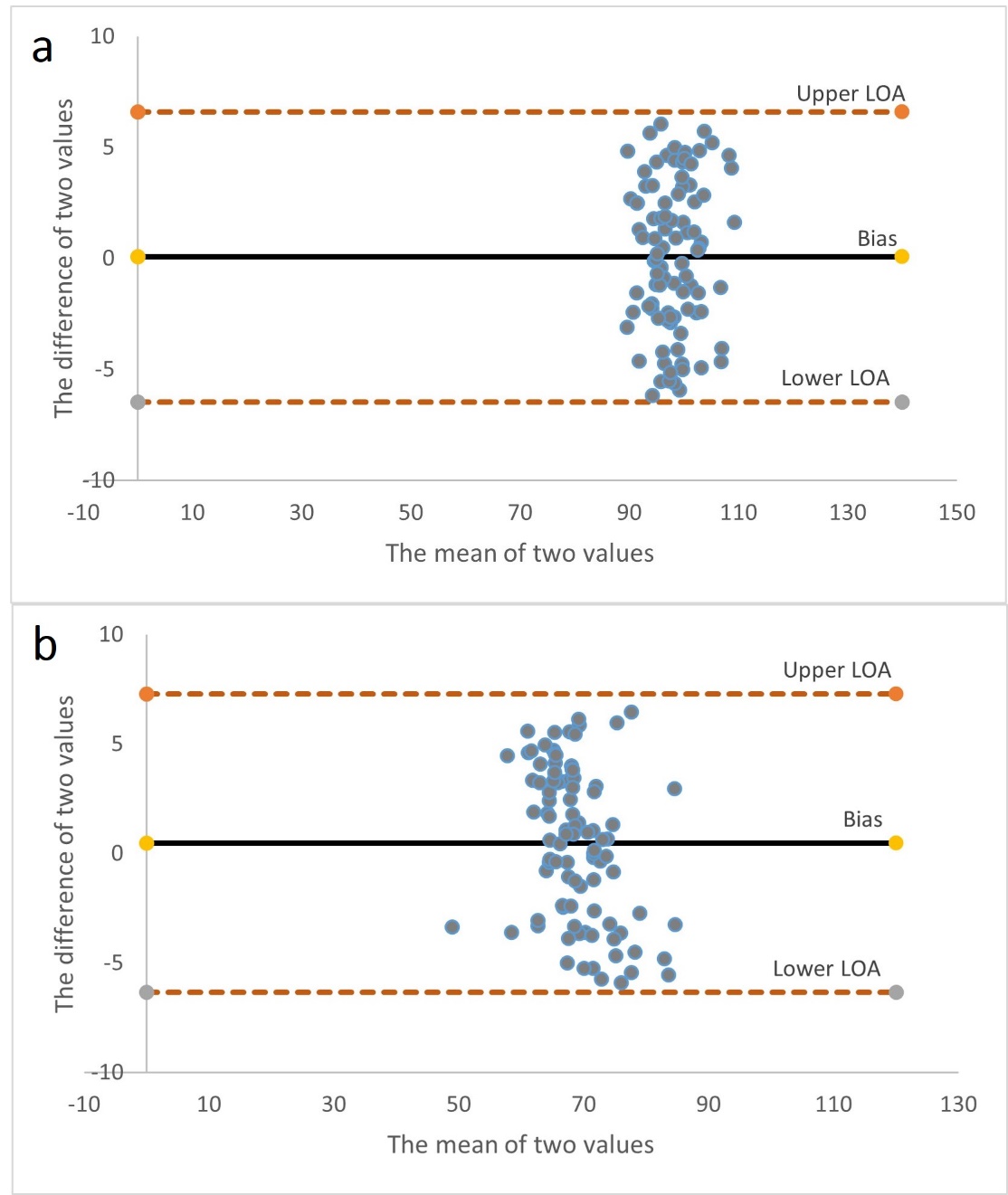


**Supplementary Figure S1.** Bland Altman plot for a) horizontal (lateral canthi distance) and b) vertical (subnasale-submental distance) measurements.


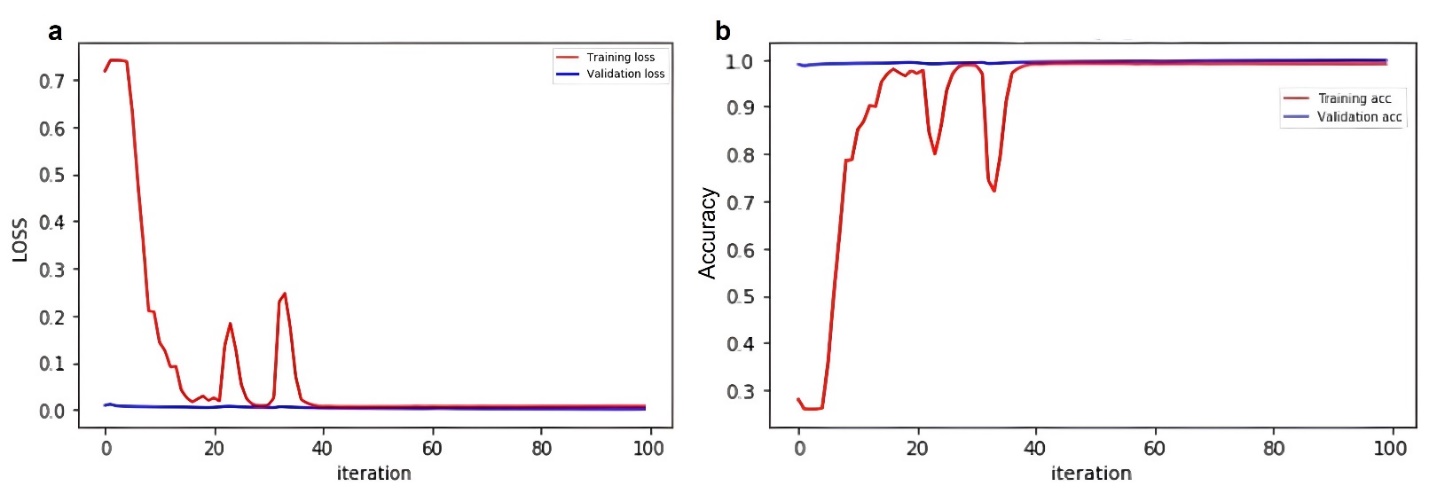


**Supplementary Figure S2.** Changes of a) training and validation loss and b) accuracy of training and validation in various epochs.
