# Supplementary material for: A deep learning framework to scale linear facial measurements to actual size using horizontal visible iris diameter: a study on an Iranian population
Source: Sci Rep. 2023 Aug 23;13:13755. doi: 10.1038/s41598-023-40839-6 (PMC10447546; doi:10.1038/s41598-023-40839-6)
Supplement: Supplementary file 3 — Supplementary Table S1. [file 41598_2023_40839_MOESM3_ESM.docx]

|  | Training Accuracy | Validation Accuracy | Training IoU | Validation  IoU | Training Jaccard distance | Validation Jaccard distance | Training Dice Coefficient | Validation Dice Coefficient |
| --- | --- | --- | --- | --- | --- | --- | --- | --- |
| Fold 1 | 99.38% | 94.83% | 99.26% | 94.53% | 0.0073 | 0.054 | 97.99% | 91.77% |
| Fold 2 | 99.80% | 99.26% | 99.76% | 99.00% | 0.0023 | 0.0099 | 99.34% | 98.38% |
| Fold 3 | 99.91% | 99.50% | 99.89% | 99.31% | 0.0010 | 0.0068 | 99.69% | 98.91% |
| Fold 4 | 99.48% | 91.57% | 99.38% | 92.36% | 0.0061 | 0.0763 | 98.52% | 90.22% |
| Fold 5 | 99.84% | 99.43% | 99.81% | 99.24% | 0.0018 | 0.0075 | 99.54% | 98.80% |
| Mean | 99.68% | 96.91% | 99.6% | 96.88% | 0.0037 | 0.0309 | 99.01% | 95.61% |
| Standard deviation | 0.236% | 3.585% | 0.281% | 3.237% | 0.00281 | 0.03227 | 0.73% | 4.258% |

**Supplementary Table S1.** Evaluation on the training & validation sets.
